# Environmentally friendly quantum-dot color filters for ultra-high-definition liquid crystal displays

**DOI:** 10.1038/s41598-020-72468-8

**Published:** 2020-09-25

**Authors:** Yun-Hyuk Ko, Prem Prabhakaran, Sinil Choi, Gyeong-Ju Kim, Changhee Lee, Kwang-Sup Lee

**Affiliations:** 1 Department of Advanced Materials, Hannam University, Yuseong-daro, Yuseong-gu, Daejeon, 34054 Republic of Korea; 2 Display Research Center, Samsung Display, Samsung-ro, Giheung-gu, Yongin-si, Gyeonggi-do 17113 Republic of Korea

**Keywords:** Chemistry, Materials science, Optics and photonics

## Abstract

This work reports the synthesis and application of highly tuned cadmium-free green and red InPZnSe_1−x_S_x_/ZnS quantum dots (QDs) in QD enhanced liquid crystal displays (LCD). The emissions of the quantum dots were synthetically tuned to sharp emissions at low full-width at half maximum. The QDs were incorporated in LCD devices as quantum dot enhancement film (QDEF) or as a quantum dot incorporated color filter (QDCF). Synthetic tuning of the gradient inter-shell in the QDs leads to reduced full width at half-maximum, resulting in sharp green and red emissions from both types of devices. The application of the same QDs to devices using these different integration techniques shows the superiority of QDCF devices over QDEF ones. The RGB color gamut of a QDCF-LCD was 81.4% of REC.2020 in the CIE 1931 color space compared to 71.2% obtained for a QDEF-LCD display. The improved performance of QDCF was mainly due to the optimal interactions between the green QDs and the green color filter. The superior performance of cadmium-free InPZnSe_1−x_S_x_/ZnS QDCFs in LCDs make them well-suited for ultra-high-definition TV formats.

## Introduction

Semiconductor quantum dots (QDs) are important emissive materials for optoelectronic applications because of their bright and well-defined emission properties. The ubiquitous potential of these materials has been established by a variety of demonstrated applications, from bio-imaging to displays and solar cells^1–3^. Despite such demonstrations and millions of dollars spent on research there are only a handful of commercialized products containing quantum dots available in the market. The reason for this is the toxicity of the elements contained in the widely studied II–VI QDs of which cadmium is a main component. Cadmium (Cd)-containing QDs are easy to synthesize and exhibit excellent optical properties. However, use of Cd-based materials are restricted in commercial products by the *restriction of hazardous substances directive* (RoHS). The safer alternative to II–VI QDs are III–V QDs based on Indium. The nucleation growth of indium (In)-based quantum dots has proven to be a difficult task because of the different nature of bonding in these materials^4^. Last decade has seen rapid advances in the understanding of III–V quantum dots, leading to the eventual commercialization liquid crystal displays (LCD) with quantum dot enhancement films (QDEF) often referred to as QLED TVs.

Quantum dot enhanced displays have high color gamut and enables ultra-high definition TV (UHDTV) formats like 4K, 8K and HDR. At the end of 2018, 4K TV technology was still being adapted by broadcasters and streaming services around the world. Currently both 8K and HDR formats suffer from lack of content due to the scarcity of terrestrial transmission of these formats. There are plans to highlight 8K TV technology during the summer Olympics in Tokyo (Tokyo 2020, *currently postponed due to covid-19 pandemic*). Widespread viewing of 8K content of this event would boost the spread of 8K technology worldwide. With 4K still going through experimental implementation in many countries, it is safe to say that we are a good part of a decade away from acceptance of 8K as a widespread broadcast format. Both these UHDTV formats with their high bandwidths are poised to dictate the visual experience and aural detail of how we will be informed and entertained in the near future^5,6^. The recent trials of 8K endoscopy equipment have demonstrated the usefulness of UHDTV formats in surgical procedures improving the level of detail accessible to the surgeon^5^.

The wider availability of UHDTV content will increase the demand for displays capable of handling their higher color gamut with a higher dynamic range. This requires new materials and fabrication strategies for displays. A solution being implemented in the market is the use of QDEF in combination with blue LED backlight unit (BLU) as a backlight in LCD displays^6^. The QDEF contains green and red QDs usually encapsulated in a poly(ethylene terephthalate) (PET) film. The green and red QDs absorb the light of the blue BLU and re-emit sharper green and red colored light, leading to a high-color gamut display^7^. Previous workers have employed Cadmium (Cd)-based, Indium (In)-based, and mixed halide perovskite QDs for QDEF layers^8–11^. The use of QDEF dramatically improves the color gamut of QD-LCD devices over conventional LCD devices. However, the proximity of quantum dots within the QDEFs are found to lead to cannibalistic absorption of light within QDEF films. Moreover, dipolar interaction between an excited green QDs with unexcited red QDs leads to fluorescent energy transfer (FRET) from green to red. Such energy transfer phenomena lead to red-biased emissions from the QDEF. This problem cannot be solved by reduction in the concentration of QDs to decrease interactions because it would lead to reduction in brightness^9^. It should be addressed by added components that help efficient light extraction from QDEF or by designing new device structures^12^.

We had recently demonstrated a new approach to enhancing the LCD display with perovskite QDs. Blue, green and red inorganic perovskite QDs were incorporated into the color filter element in the LCD to form a functional quantum dot incorporated color filter (QDCF)-to achieve lower overlap between RGB color elements in pixels^8^. Lead halide perovskites has high-intensity emissions with narrow *full width at half-maximum* (FWHM)^13^. However, lead halide perovskite QDs are unsuited for mass market applications because of their instability, and presence of lead (Pb) in them^14^. Indium-based quantum dots are better suited for commercial applications but their widespread application has been hampered due to the difficulty in synthetically controlling their complex crystallization behaviour and optical properties^4,15^.

**Figure 1:**
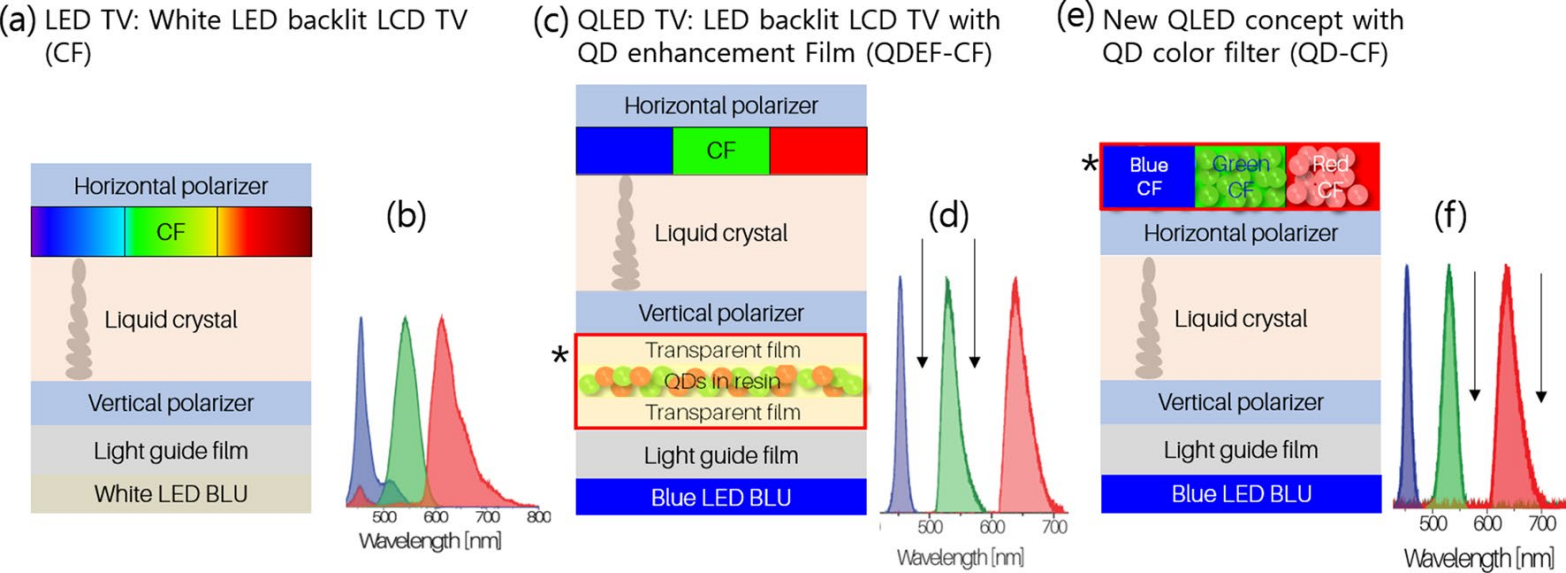
Different paradigms in LCD display. (**a**) White LED backlit LCD display also known as LED-TV. (**b**) Emission characteristics of the device in (**a**). (**c**) Blue LED backlit LCD display with QD enhancement film also known as QLED-TV. (**d**) Emission characteristics of the device in (**c**). (**e**) QD-color filter (QDCF) for QLED-TV. (**f**) Emission characteristics of the device in (**e**). The star symbol (*) indicates newly introduced parts of the device compared to preceding device. Each arrow (↓) in a spectrum indicates regions where the extent of overlap between PL emissions have been reduced compared to the previous spectrum.

Here we demonstrate the customization of PL emissions from environment friendly InPZnSe_1−x_S_x_/ZnS QDs and their use in QDCFs. The QDCF has green and red QDs specifically synthesized to have emissions with reduced FWHM, leading to less overlap and wider coverage of the Rec.2020 color gamut. A summary of the concept of this work is presented in Fig. 1, where different types of devices and their polarized emissions are presented. A white LED backlit LCD display with a conventional color filter (CF) (here in after mentioned as LCD) is shown in Fig. 1a. There is a lot of crosstalk between the blue, green and red emissions in this device, as seen in the emission spectrum shown in Fig. 1b. The second device in Fig. 1c constitutes a blue LED backlit LCD display with a quantum dot enhancement film (QDEF), called QDEF-LCD from here on. This device features sharp emissions with decreased FWHM due to the introduction of QDEF in the device leading to reduced crosstalk between blue, green, and red emissions, as seen in the corresponding spectrum in Fig. 1d. The QDEF is made by QDs encapsulated in a polymer resin at high concentrations. The third device structure is our recently proposed paradigm for QLED devices^8^. It involves emission-tuned green and red InPZnSe_1−x_S_x_/ZnS QDs mixed with green and red color filters creating a quantum dot incorporated color filter (QDCF) element. Here QDCF substitutes the role of QDEF in the display, leading to an overall increase in emission intensity of the device while contributing to sharper green and red emission. This third type of device will be called QDCF LCD from here on. The device structure can be seen in Fig. 1e and the characteristic emission can be seen in Fig. 1f.

The interplay of components such as the backlight unit (BLU), the color filters, QDEF/QDCF, etc. in the display dictates the color and brightness delivered by the variants of LCD displays featured in Fig. 1. Color filters transmit a single color (red, green, or blue) from the emissions passing through them. A wide color gamut and high dynamic range results from the tandem action of both the emissive components and color filters. Overlaps between PL emissions of blue, green, and red emissions give rise to crosstalk and reduces color gamut. In this work, the emissive properties of environmentally friendly InPZnSe_1−x_S_x_/ZnS QDs have been customized such that their incorporation in color filters leads to sharp, non-overlapping emissions and hence a wide color gamut.

## Results and discussion

**Figure 2:**
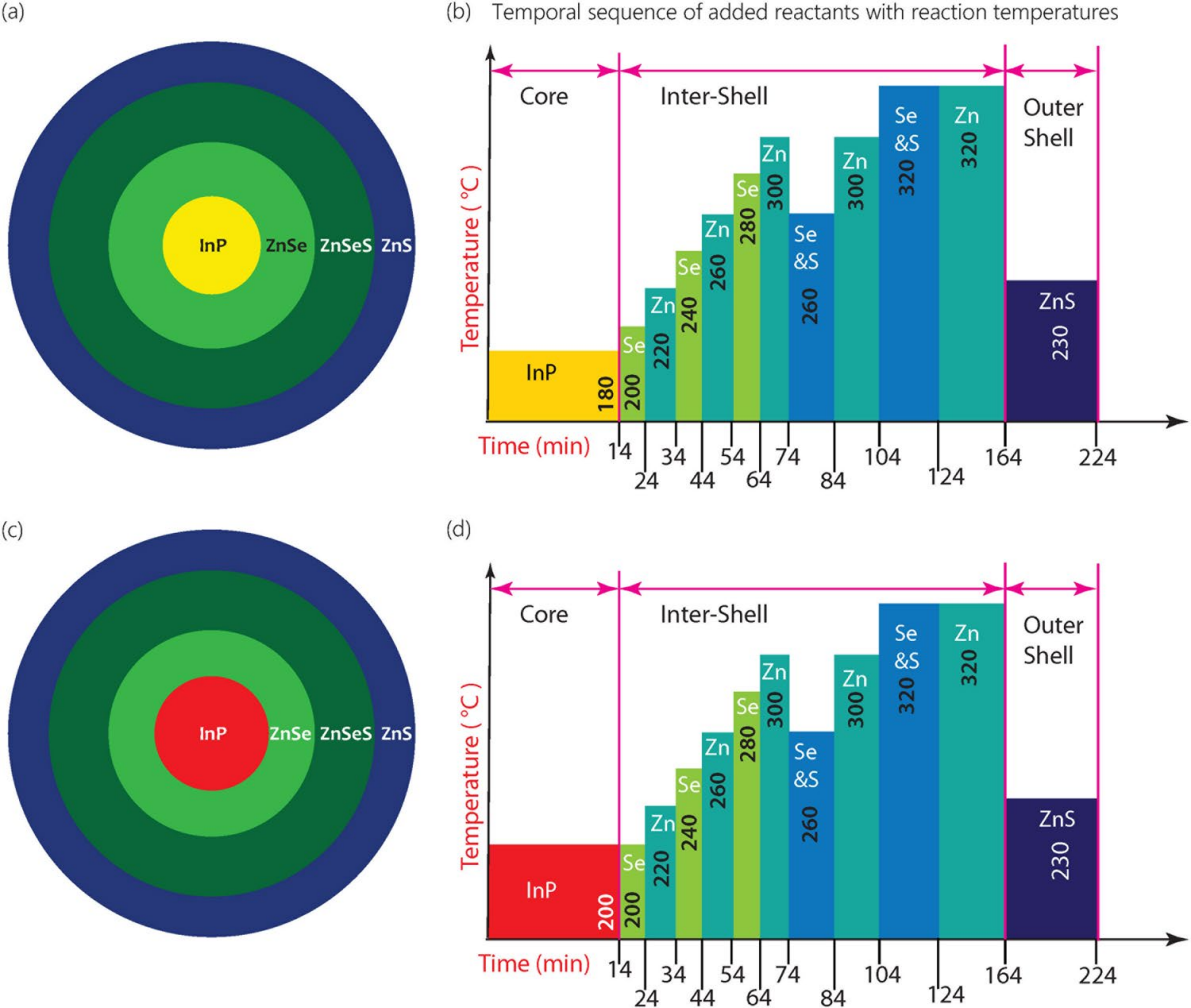
A summary of steps during the synthesis of InP/ZnSeS/ZnS QDs. (**a**) Illustration of composition gradient in the green InP/ZnSeS/ZnS (GQD) core/shell structure. (**b**) The temporal sequence of addition of different materials with corresponding temperatures during the synthesis of green QDs. (**c**) Illustration of composition gradient in the red InP/ZnSeS/ZnS (RQD) core/shell structure. (**d**) The temporal sequence of addition of different materials with corresponding temperatures during the synthesis of red QDs.

Environmentally friendly green quantum dot (GQD) and red quantum dot (RQD) with a generic core/shell structure of InPZnSe_1−x_S_x_/ZnS were synthesized for this work. The InP-based QDs (InP QDs) showed a high photoluminescence-quantum yield (PL-QY) and a narrow FWHM, as well as tunable absorption and emission properties to suit the peak transmitted wavelengths of a color filters used in LCDs. These QDs were synthesized by tuning the In^3+^:P^3−^ stoichiometric molar fraction in the core (of the QDs) to absorb the blue light from the LED backlight unit and emit only green, and red-light via energy-down-shift^16^. The QDs were synthesized by the successive ionic layer adsorption and reaction (SILAR) method. The summary of steps during the synthesis of green and red InPZnSe_1−x_S_x_/ZnS QDs is given in Fig. 2. The growth temperature for GQD and RQD cores was 180 °C and 200 °C, respectively. The growth of the shell in both green and red QDs follows the same procedure involving the introduction of different ions at different temperatures, as seen in Fig. 2b,d. Such additions lead to a shell with a gradient concentration of Se moving from a Se-rich ZnSe on top of the core to an S-rich ZnS outer layer through an intermediate layer (interlayer) of gradually varying Se and S content. Hence, the inner layer and gradient layer compositions are expressed together as ZnSe_1−x_S_x_.

The difference in energy level between the core and the shell determines the quantum yield of fluorescence, while the extent of a lattice mismatch determines the FWHM of its emission spectrum. The gradient shell structure helps reduce the lattice mismatch within the core/shell structure^17,18^.

**Figure 3:**
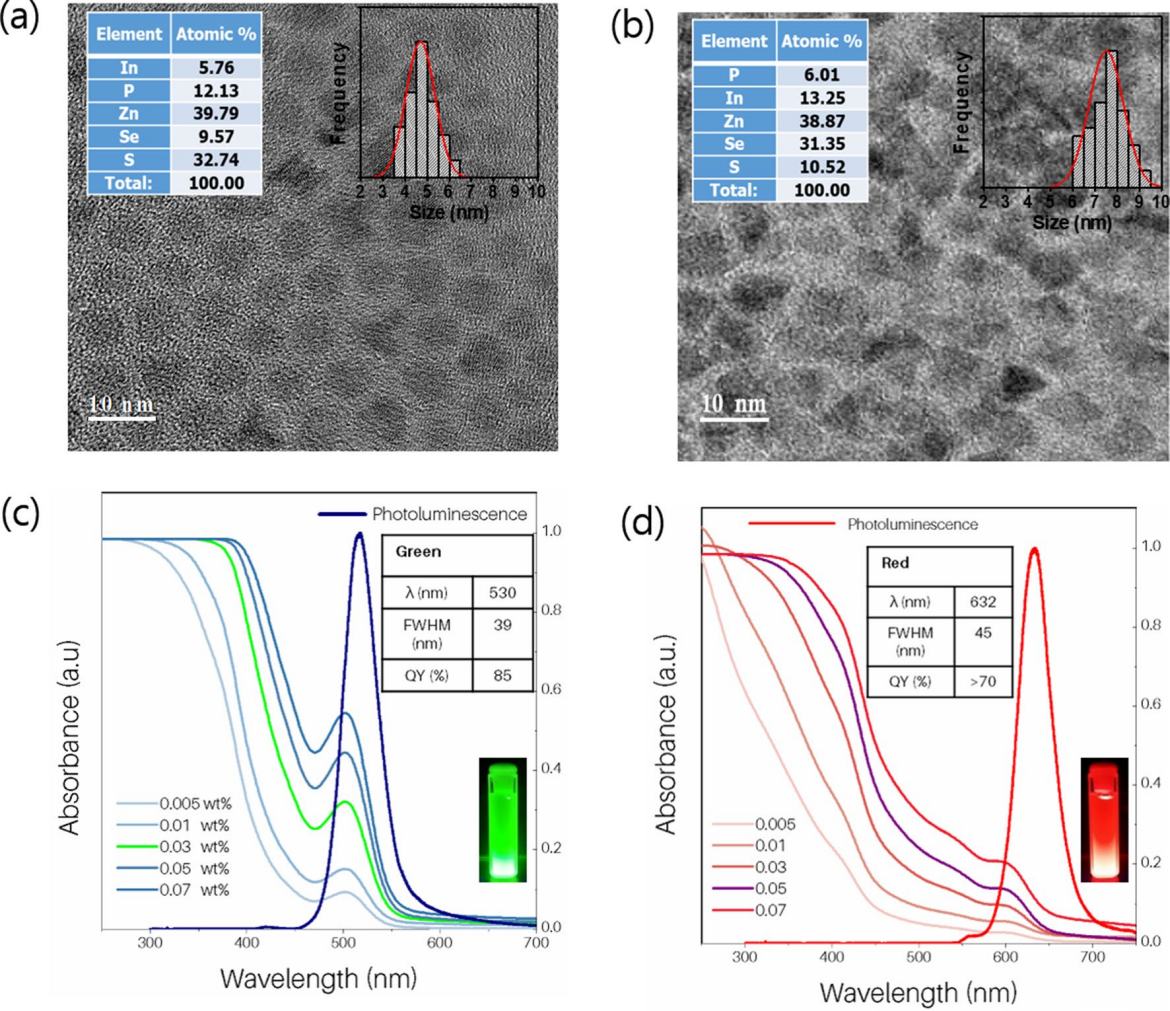
(**a**) TEM image of green QDs with size distribution. The inset summarizes the optical properties of the GQD. (**b**) TEM image of red QDs with size distribution. The inset summarizes the optical properties of the RQD. (**c**) Absorption spectrum corresponding to different concentrations of GQD nanoparticle dispersion, where the sharp curve on the right is the fluorescence spectrum. (**d**) Absorption spectrum corresponding to different concentrations of RQD nanoparticle dispersion. The inset summarizes the optical properties of the RQD, where the sharp curve on the right is the fluorescence and summarizes the optical properties of the RQD, and the sharp curve on the right is the fluorescence spectrum.

Both GQD and RQD were highly crystalline with low polydispersity, as seen in the HR-TEM images in Fig. 3a,b. The QDs had a cubical shape, unlike the Cd or InP-based core/shell QDs (i.e., spherical-like) commonly used in QDEF LCD^16,19,20^. The average sizes of GQD and RQD were 5.12 nm and 7.07 nm, respectively. The GQD and RQD showed respective lattice constants of 5.8 and 5.9 Å. The composition of the QDs determined by energy dispersive X-ray spectrometry (EDS) measurement are shown in the insets of Fig. 3a,b. InP QDs have a zinc blende crystalline structure (JCPDS No. 32-0452)^21^, which was confirmed by selected area electron diffraction (SAED) and X-ray diffraction (XRD) patterns (see Supplementary Figures S4a–d in the supporting information). The distinct rings in the SAED spectrum originating from the 111, 220, and 311 planes is quite consistent with the dominant XRD diffraction peaks for GQDs and RQDs. The larger size of RQDs leads to sharper XRD peaks compared to GQDs.

Note that gradient shell GQDs and RQDs described here have complex internal structures which could only be thoroughly characterized techniques such as X-ray photoelectron spectroscopy (XPS) or X-ray absorption fine structure study (XAFS)^22,23^. QDs are quite difficult to analyse with powder X-ray diffraction because of the small size of the nanoparticles effecting broadening of the spectrum. XPS has been used to study the extent and type of alloying in quantum dots by analysing the energies of the photoelectrons^23^. XAFS is more suitable for studying nanocrystals because it is independent of long-range ordering in crystals^22^.

The optical properties of GQD and RQD are summarized in Fig. 3c,d. The UV–vis absorption characteristics of the QDs were recorded at different concentrations ranging from 0.005 to 0.07 wt%. Within this range of concentrations, the absorption peak intensities were found to increase with increasing concentration. The PL peak presented in both cases corresponds to a dispersion with a concentration of 0.07 wt%. The emission spectrum of the GQD showed an emission maximum at 530 nm with an FWHM of 39 nm and quantum yield (QY) greater than 85%. The RQDs showed an emission maximum at 632 nm with an FWHM of 45 nm and quantum yield (QY) greater than 70%. The quantum yields were determined using time-resolved PL decay experiments. The QY for GQD and RQD was calculated from exciton lifetimes. The detailed calculation is summarized in Supplementary Figure S5 and Supplementary Table 1 in the supporting information.

**Table 1:** Optical characteristics of LCD, QDEF-LCD and QDCF-LCD.

Sample	Peak frequency	PL peak WL (nm)	FWHM (nm)	CIE coordinates (x,y)	Color gamut	
					NTSC[%]	Rec. 2020[%]
Conventional LCD	Blue	456	20.6	(0.14, 0.10)	73.7	55.1
	Green	541	49.1	(0.29, 0.68)		
	Red	612	58.7	(0.61, 0.33)		
QDEF LCD	Blue	452	20.4	(0.15, 0.03)	95.2	71.2
	Green	528	31.3	(0.24, 0.70)		
	Red	638	36.4	(0.64, 0.30)		
QDCF LCD	Blue	452	20.4	(0.15, 0.03)	108.8	81.4
	Green	530	26.1	(0.18, 0.74)		
	Red	638	36.7	(0.65, 0.30)		

**Figure 4:**
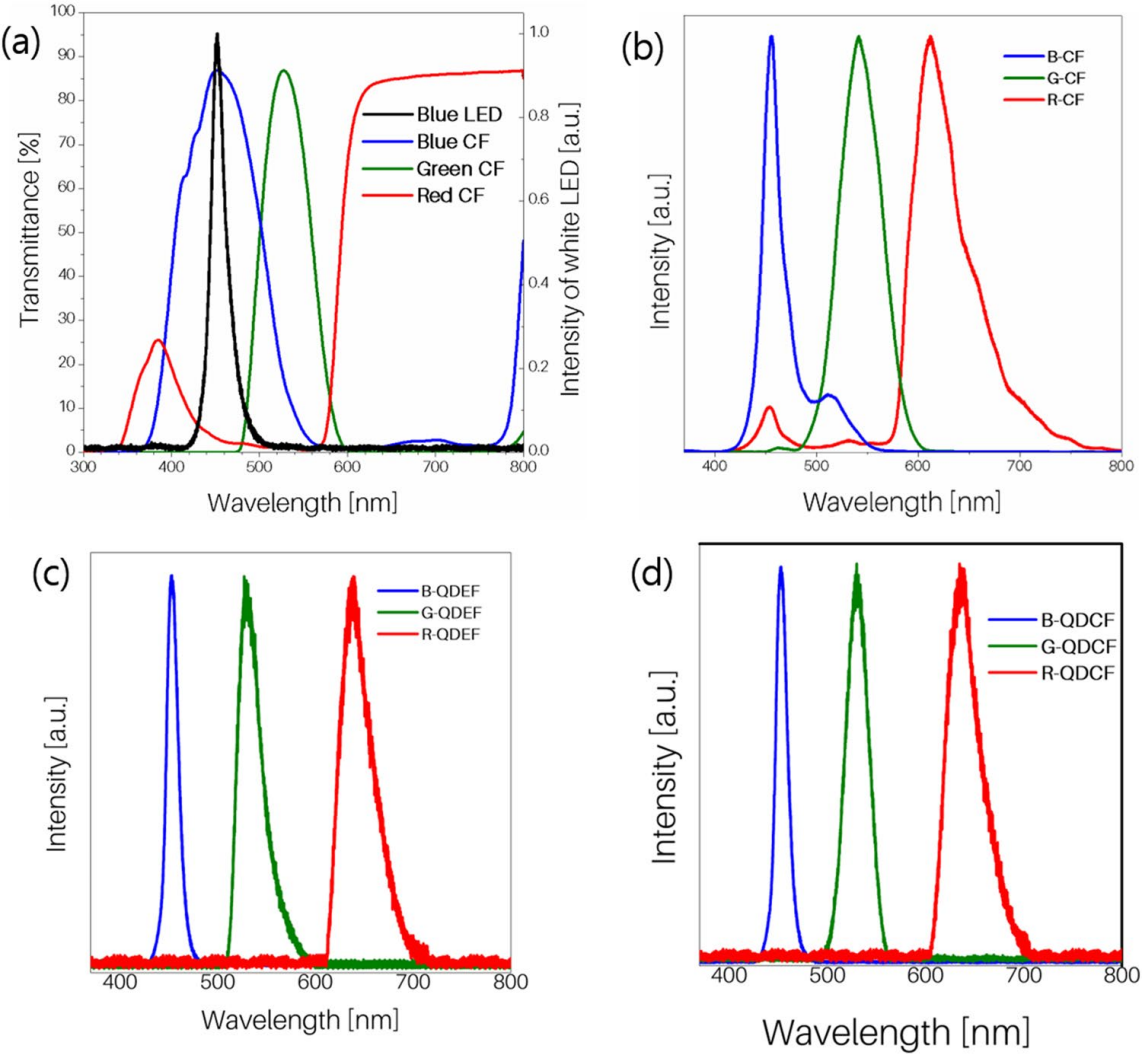
Optical characteristics of various components constituting the fabricated displays. (**a**) Emission properties of the BLU and color filter (sharp black curve) to the percentage transmittance of blue, green and red color filters. RGB emission of (**b**) white LED Backlit LCD (**c**) BLU backlit LCD device with QDEF (**d**) BLU backlit LCD device with quantum dot integrated color filters (QDCF).

The best color gamut performances are achieved in LCD devices when the blue, green, and red emissions fall well within the range of wavelengths transmitted by the blue, green and red color filters. Minimal spectral overlap between emissions after transmission through the color filters is crucial to the efficiency of these devices. In the case of the quantum dot-enhanced LCD devices discussed in this work, the blue emission originates from the blue LED backlight unit (BLU). The emissive characteristics of the BLU, giving rise to an emission maximum of 452 nm as seen in Fig. 4a. This was measured by arranging a blue LED from the BLU in the PL spectrometer. The emission spectrum of the blue color filter was measured by coating it on a quartz slide and using it as a sample to measure fluorescence. The same procedure using the quartz substrate was followed in the case of green and red color filters. The range of wavelengths transmitted by the blue (371–563 nm, λ_max_ = 451 nm), green (478–595 nm, λ_max_ = 527 nm) and red (> 570 nm, λ_max_ = 631 nm) color filters used in LCD devices are represented by the blue, green and red curves in Fig. 4a, respectively.

As mentioned earlier, three different LCD device structures were fabricated for this study, namely LCD, QDEF-LCD and QDCF-LCD. The first one is a white LED BLU-lighted LCD display with a conventional device structure, as seen in Fig. 1b; the second one, QDEF-LCD, is a blue BLU-lit QD-enhanced LCD with QDEF, as seen in Fig. 1c; and the third one, QDCF-LCD, is a blue backlit QD-enhanced LCD with a QDCF, as shown in Fig. 1d. The conventional LCD device gave polarized blue emissions at 456 nm with an FWHM of 20.6 nm, the green emission had a peak wavelength of 541 nm with an FWHM of 49.1 nm. The red color polarized fluorescence from the above device was at 612 nm with an FWHM of 58.7 nm. The PL emission of the conventional LCD device given in Fig. 4b. shows considerable polarized PL spectrum crosstalk (overlap) between red, green and blue emission spectra. The spectral characteristics are summarized in Table 1.

The polarized blue, green and red emissions from the QDEF-LCD can be seen in Fig. 4c. The polarized blue emission from QDEF-LCD device peaked at 452 nm with an FWHM of 20.4. The polarized green emission peaked at 528 nm with an FWHM of 31.3 nm. The polarized red emission peaked at 638 nm with an FWHM of 36.4 nm. In Fig. 4c, the polarized PL emissions from the QDEF-LCD are almost free of crosstalk because there is little or no overlap between the emissions. This leads to a considerable improvement in the color gamut of the LCD.

The QDCF-LCDs were fabricated by integrating GQD and RQD into green and red color filters, respectively. The direct integration of quantum dots into the color filters means that QDCF-LCD devices does not need a QDEF (compare devices in Fig. 1c,e). The emission wavelength (λ_max_) and FWHM governs the color gamut of a display^24^. Incorporation of QDs into devices as QDCFs increases the color gamut by improving the fwhm of GQDs. The polarized blue, green and red PL emissions from the QDCF-LCD can be seen in Fig. 4d. The blue emissions peaked at 452 nm with an FWHM of 20.4 nm. The green emissions peaked at 530 nm with an FWHM of 26.1 nm. The red emissions peaked at 638 nm with an FWHM of 36.7 nm. In the tabulated FWHM data in Table 1, green and red emissions in the QDCF-LCD shows a considerable reduction compared to the conventional LCD device. The blue emission of the QDEF-LCD and the QDCF-LCD was slightly blue-shifted compared to the LCD display because of the difference in backlight units. The blue emissions from all three devices show similar narrow FWHM of around 20 nm. The FWHM of the green emission decreases in the QDCF-LCD compared to the QDEF-LCD, leading to the possibility of a wider color gamut in the former. The emission maximum of the GQD in solution is 530 nm. When the GQD is integrated into the green color filter in the QDCF-LCD the range of wavelengths available for the QD is controlled by the wavelength filtering action of the color filter compound (478–595 nm, λ_max_ = 527 nm). Hence, the combination emits at a peak wavelength of 530 nm. The red shift in green emission peaks of the QDCF-LCD compared to the QDEF-LCD may be due to the restriction on absorption wavelengths placed by the color filter. The absorption spectrum of the GQD in solution is compared with the transmitted wavelengths of a green color filter and the emission of a QD integrated green color filter in Supporting Figure S6(a). Similarly, the red color filter restricts the incoming wavelengths available for the red QDs in the red QDCF to wavelengths greater than 570 nm. The emissions peak of RQD incorporated into QDCF is at 638 nm. The red emission maximum in the QDCF-LCD is the same as the polarized red emission from the QDEF-LCD. The absorption spectrum of the RQD in solution is compared with the transmitted wavelengths of the red color filter and the emission of the QD integrated red color filter in Supporting Figure S6(b).

**Figure 5:**
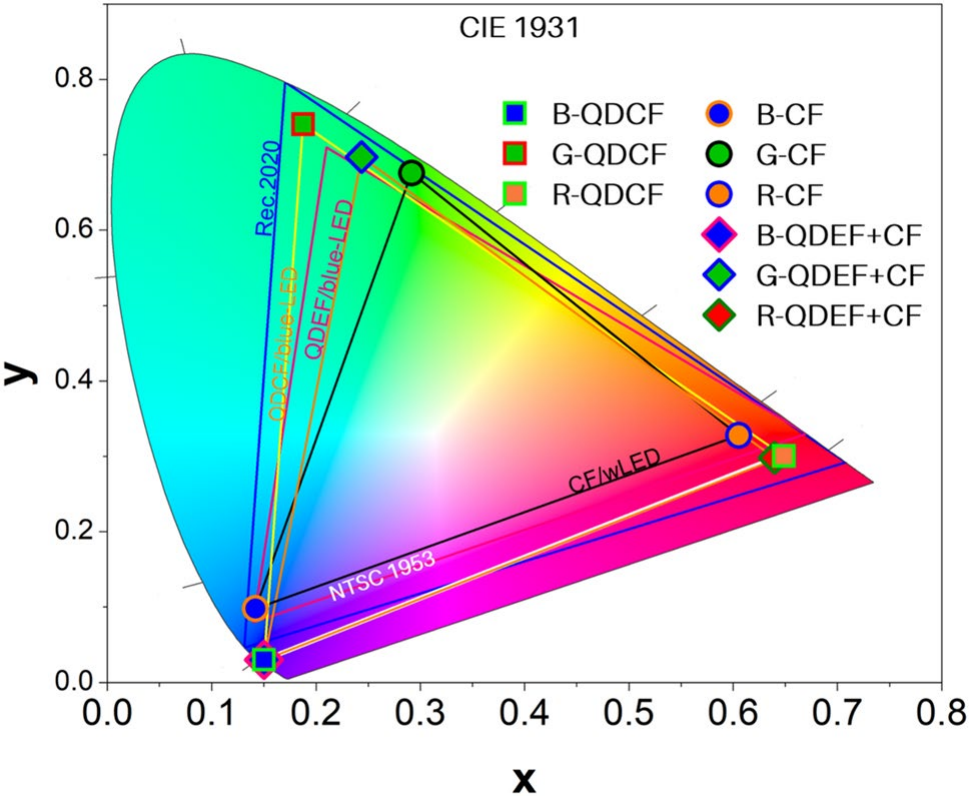
Comparison of the RGB color triangles formed by the CIE (CIE 1931) coordinates of blue, green, and red polarized PL emissions of conventional LCD, QDEF-LCD and QDCF-LCD. The color gamut defined by the NTSC 1953 standard is represented as a pink triangle, while the color gamut of Rec.2020 standard is represented by the blue triangle.

The color gamut performance of the three different displays was studied in order to assess their potential as ultra-high-resolution LCD displays. The performances of LCD, QDEF-LCD and QDCF-LCD were compared by estimating their coordinates in the CIE 1931 color space using two different standards, namely NTSC 1953 and Rec.2020. Among these, the specification for UHD displays falls in the Rec.2020 color space. The CIE coordinates of the three types of LCDs were calculated from their PL spectra in Fig. 4b,d and a MATLAB-based CIE coordinate calculator. The calculated CIE coordinates for all devices are summarized in Table 1. The two standard color spaces describe triangular graphs in the CIE color space, as seen in Fig. 5. The triangle of a standard in the CIE space encompasses all colors that could be expressed by a display that complies with that standard.

The RGB color gamut of the conventional LCD is 73.7% of the NTSC standard and 55.1% of the Rec. 2020 standard. The coordinates calculated from the emission of a conventional LCD are shown as closed circles. The triangle formed by the coordinates of the LCD in the blue, green and red regions of the color space fills only 73.7% of the NTSC standard color space and further falls short by covering only 55.1% of the Rec.2020 color space. This means that this display is more suited for standard resolution displays than UHD displays. The CIE co-ordinates of the QDEF-LCD are represented as diamond (◊) symbols in Fig. 5. The QDEF-LCDs cover respectively cover 95.2% of the NTSC colorspace and 71.2% of the REC 2020 color space. The standard wavelength corresponding to blue, green, and red colors in REC.2020 standard are 467 nm, 532 nm and 630 nm respectively. The emissions of the QDEF-LCDs corresponding to blue, green, and red are 452 nm, 528 nm and 638 nm respectively. Previous studies comparing the performance of red InP QDs have established that they show high REC.2020 color gamut performance when the red emission was at 652.1 nm; when the red wavelength is decreased to match the standard 630 nm the gamut performance fell well below 90%^10^. Hence improving the performance of red color is important in improving the REC.2020 performance of the device. Such an improvement would result from closely understanding the interaction between the QD emissions and the color filter and optimizing their interaction. However, when QDs were incorporated in the device in the form of QDCF an improved performance of 108.8% in the NTSC color space and 81.4% of REC 2020 colorspace was obtained. This broad coverage offered by QDCF in both NTSC and REC 2020 colorspaces enable QDCF LCDs to express greater color and dynamic range compared to LCDs and QDEF LCDs. We have earlier reported an RGB color gamut of 100.4% for NTSC standard and 134.2% for REC 2020 standard for QDCF LCD devices based on perovskites^8^. In another work involving perovskite QDs Lin and co-workers reported 158.93% of NTSC and 118.60% of Rec. 2020 respectively^25^. However, there are no reports of QDCF type devices with InP based quantum dots showing high performances described here. The dramatic enhancement in the green emission in QDCFs compared to QDEF results from the synergistic action of the green color filter and the GQD emissions. The excitation wavelengths available from the GQDs in the QDCF are expressed by the overlap of the GQD absorbance spectrum and the transmission spectrum of green color filter in Figure s6a. This overlap results in a sharp green emission (530 nm) close to the Rec.2020 green wavelength standard 532 nm. The red emission from the QDCF device (638 nm) deviates to a greater extent from the Rec.2020 red wavelength standard 630 nm. The red color emission also shows broader FWHM due broader overlap between the red color filter and absorption of the RQD seen in Figure S6b. Developing a better understanding of the interaction between the QDs and the QDCFs will lead to even better devices of this genre in the future.

## Conclusions

Synthesis of highly bright cadmium-free red and green InPZnSe_1−x_S_x_/ZnS QDs and their application in UHD LCDs have been demonstrated in this work. The synthetic tuning of QDs with gradient interlayer (ZnSe_1−X_S_X_) leads to highly bright QDs. The green QD has a quantum yield of 85% (FWHM 26.1 nm) and the red QD has quantum yield of 70% (FWHM 36.7) respectively. The use of QDs in QDCFs ushers in the third paradigm shift in LCD technology. It overcomes the limitations of QDEFs like the loss of optical power due to encapsulation driven interaction of QDs. The apparent optical output from QDCFs also depend on the color filter materials because the color filter governs the wavelengths accessible during QD excitation by blue backlight. The color gamut performance of the QDCF LCDs improves due to the reduced FWHM of green QDs. Crucially high color gamut is perceived as brightness by the human eye due to Helmholtz–Kohlrausch Effect^26^. We believe that further investigation to understand the interaction between the red QD and the corresponding color filter could lead to improved red emissions and push the QDCF devices towards full REC 2020 compliance.

## Supplementary information

Supplementary file1

## Data Availability

All data generated or analysed during this study are included in this published article (and its Supplementary Information files). Further data associated with publication is available from the authors upon reasonable request.
