# Supplementary material for: Environmentally friendly quantum-dot color filters for ultra-high-definition liquid crystal displays
Source: Sci Rep. 2020 Sep 25;10:15817. doi: 10.1038/s41598-020-72468-8 (PMC7519667; doi:10.1038/s41598-020-72468-8)
Supplement: Supplementary file 1 — Supplementary file1 [file 41598_2020_72468_MOESM1_ESM.docx]

**Supplementary Information**

**Environmentally Friendly Quantum-Dot Color Filters for**

**Ultra-High-Definition Liquid Crystal Displays**

Yun-Hyuk Ko,^1,2#^ Prem Prabhakaran,^1#^ Sinil Choi,^1^ Gyeong-Ju Kim,^1^ Changhee Lee,^2*^ Kwang-Sup Lee^1*^

*^1^Department of Advanced Materials, Hannam University, Yuseong-daro, Yuseong-gu, Daejeon 34054, Republic of Korea*

*^2^**Display Research Center, Samsung Display, #1 Samsung-ro, Giheung-gu, Yongin-si, Gyeonggi-do 17113, Republic of Korea*

*Corresponding authors: e-mail: [chlee7@samsung.com](mailto:chlee7@samsung.com), phone +82-31-5181-3100;

e-mail: [kslee@hnu.kr](mailto:kslee@hnu.kr), phone +82-42-629-8857;

^#^ These authors contributed equally to this work.

**Materials and Methods**

***Materials*:** Indium(III) iodide (InI_3_, 99.998%), zinc(II) chloride (ZnCl_2_, 99.999%), zinc(II) iodide ZnI_2_, 99.99%), tris(diethylamino)phosphine ((DEA)3P, 97%), selenium (99.99%, powder 100 mesh), sulfur (trace metals basis 99.998%), 1-dodecanethiol (DDT, ≥98%), zinc acetate (Zn(OAc)_2_, 99.99%), zinc stearate (ZnSt_2_, technical grade 65%) trioctylphosphine (TOP, 97%), oleylamine (OLA, 70 %), and octadecene (ODE, technical 90%) were purchased from Sigma-Aldrich. Dual-component thermally curable silicone resin (OE-6630 A and B) was purchased from Dow Corning. R, G, and B color filters (model numbers: DCR-TR711R, DCR-TR711G, and DCR-TR711B) were purchased from Dongjin Semichem Co.

***Preparation of Stock Solution:*** A selenium and trioctylphosphine (SeTOP) stock solution was prepared by the dissolution of 1.78 mmol of Se powder in 1 mL of TOP. A selenium, sulfur, and trioctylphosphine (SeSTOP-1) stock solution was prepared by the dissolution of 0.06 mmol of Se powder and 2 mmol sulfur powder in 2 mL of TOP. A selenium, sulfur, and trioctylphosphine (SeSTOP-2) stock solution was prepared by the dissolution of 0.02 mmol of Se powder and 4 mmol sulfur powder in 2 mL of TOP. For zinc streate (ZnSt_2_) stock solution, 4.74 mmol of ZnSt2 and 15 mL ODE were mixed in a 150 mL three-neck flask. This mixture was heated to 120 °C and degassed and dried under vacuum at 120 °C for 1 h. Then, the temperature was raised to 250 °C under N_2_. The ZnSt_2_ stock solution was stored in a three-neck flask under N_2_ and this temperature.

***Synthesis of green and red gradient shell InP/ZnSe_x_S_1-x_/ZnS QDs:*** 0.111 g (0.225 mmol) of indium(III) Iodide, 0.15 g (1.1 mmol) of zinc(II) chloride, 0.351 g (1.1 mmol) of zinc(II) Iodide were mixed in 6.0 mL of technical oleylamine, which is a coordinating solvent. The reaction mixture was stirred and degassed at 150 °C for an hour, and then heated to 180 °C under inert atmosphere for green QDs. In the case of red QDs, this temperature was raised to 200 °C. Upon reaching the target temperature, a volume of 0.5 mL (2.28 mmol) of tris(diethylamino)phosphine ((DEA)3P) (phosphorus: indium ratio=6.5:1) was swiftly injected in the above mixture. After the phosphorus precursor injection, the growth of an InP core proceeded at that temperature for 14 min.

The successive growth of the ZnSeS intermediate shell was performed by alternate injection of anionic and cationic shell precursors at appropriate shelling durations and designated temperatures as follows. After the mixture of 0.09 mL of SeTOP stock solution was injected, this reaction mixture was maintained at 200°C for 10 min. After the mixture of 0.5 mL of ZnSt_2_ stock solution was slowly injected, this mixture was maintained at 220°C for 10 min. After the mixture of 0.09 mL of SeTOP stock solution was injected, this mixture was maintained at 240 °C for 10 min. After the mixture of 0.5 mL of ZnSt_2_ stock solution was slowly injected, this mixture was maintained at 260 °C for 10 min. After the mixture of 0.09 mL of the SeTOP stock solution was injected, this mixture was maintained at 280 °C for 10 min. After the mixture of 0.5 mL of ZnSt_2_ stock solution was slowly injected, this mixture was maintained at 300 °C for 10 min. The mixture was cooled down to 260 °C, and the mixture of SeSTOP-1 stock solution was injected at this temperature and maintained for 10 min. After the mixture of 6 mL of ZnSt_2_ stock solution was slowly injected, this mixture was maintained at 300 °C for 20 min. After the mixture of 2 mL of SeSTOP-2 stock solution was slowly injected, this mixture was maintained at 320 °C for 20 min. After the mixture of 7 mL of ZnSt_2_ stock solution was slowly injected, this mixture was maintained at the same temperature for 40 min. For the ZnS shell passivation, the reaction flask was cooled down to room temperature and 0.22 g (1.2 mmol) of Zn(OAc)_2_ was added followed by heating up to 150 °C. At 150 °C dodecanethiol 0.48 mL (2 mmol) of was added to the reaction flask and heated to 230 °C for 1 h to complete the surface passivation. After an hour, the reaction mixture was cooled down to room temperature. To purify the crude product, 2 mL of chloroform, 1 mL of ethanol, and an excess amount of acetone were added to the resulting solution until the solution became turbid, which was precipitated by centrifugation at 10,000 rpm. After centrifugation, the supernatant was decanted and the precipitate was re-dispersed in organic solvents such as chloroform, toluene, and hexane. It was then dispersed in an organic solvent for the following optical characterization and device fabrication. The synthesis of quantum dots is summarized in Supplementary Figures S2 and S3 in the supporting information.

***Measurements:*** The detailed morphology, size distribution, composition ratio, and crystal structure of QDs were characterized by a transmission electron microscope (TEM) with an acceleration voltage of 200 kV, energy dispersive X-ray spectroscopy (EDS), and X-ray diffraction (XRD). UV-visible absorption spectra were recorded with a UV-3600 (Shimadzu, Japan). The photoluminescence (PL) spectra, its characteristics and absolute quantum yields were recorded using a QE-2100 (Otsuka Electronics). In addition, the exciton lifetime for QDs was confirmed by measuring time-resolve PL decay curves (Hamamatsu C11347-11 using 365-nm LED source and 1 MHz).

***Preparation of color filters:*** Commercial color filter (CF) compounds marketed by Dongjin Semichem Co. were used. The red, green and blue color filter compounds used were DCR-TR711R, DCR-TR711G, and DCR-TR711B, respectively. A 1 cm^2^ quartz substrate was immersed in a 1:1 solution of isopropanol and acetone and sonicated for 10 mins. It was dried under a stream of N_2_ gas. Guide rings of dimensions 1 cm × 1 cm were attached to the dried quartz substrate and blue, green and red color filters were coated uniformly on it. The coated quartz substrates were dried at room temperature to harde the film.

***Fabrication of QD enhancement film (QDEF):*** Stock solutions of green and red quantum constituting individual QDs were dispersed in hexane at 20 wt%. A composite resin was prepared for making QDEF by mixing 0.3 mL of GQD and RQD dispersions in hexane with a silicon resin. Guide rings of dimensions 1 cm × 1 cm were attached to the dried quartz substrate and the composite resin was coated evenly on the substrate. This was followed by drying the film at room temperature to harden it.

***Fabrication of QD functional CFs (QDCF):*** Green and red color filter compounds were mixed with 0.3 mL (20 wt%) GQD and RQD solutions in a glass vial. The mixture of red CF with red QD, green CF with green QD and pristine blue CF was coated uniformly employing guide rings on a 1 cm^2^ quartz as mentioned above and dried in ambient conditions to make a hardened film.

***Fabrication and characterization of LCD devices:*** The LCDs had the stacked structures seen in Figure 1(a,c,e). For the conventional LCD device, a white LED was used as the backlight unit (BLU) while a blue LCD was used as a BLU for both quantum dot incorporated LED devices. The LCD device and the QDCF-incorporating LCD device had the device structure of BLU/light guide film/vertical polarizer/nematic liquid crystal/horizontal polarizer/CF. In the case of QDCF-LCD, the QDCF element replaced the CF. Both QDCF and QDEF films were kept at 500 µ thickness. The QDEF device incorporates the QDEF film between the light guide film and the vertical polarizer, as seen in Figure 1(c). The polarized PL emission of the devices and the FWHM was measured using a polarizer microscope (Nikon Inc).


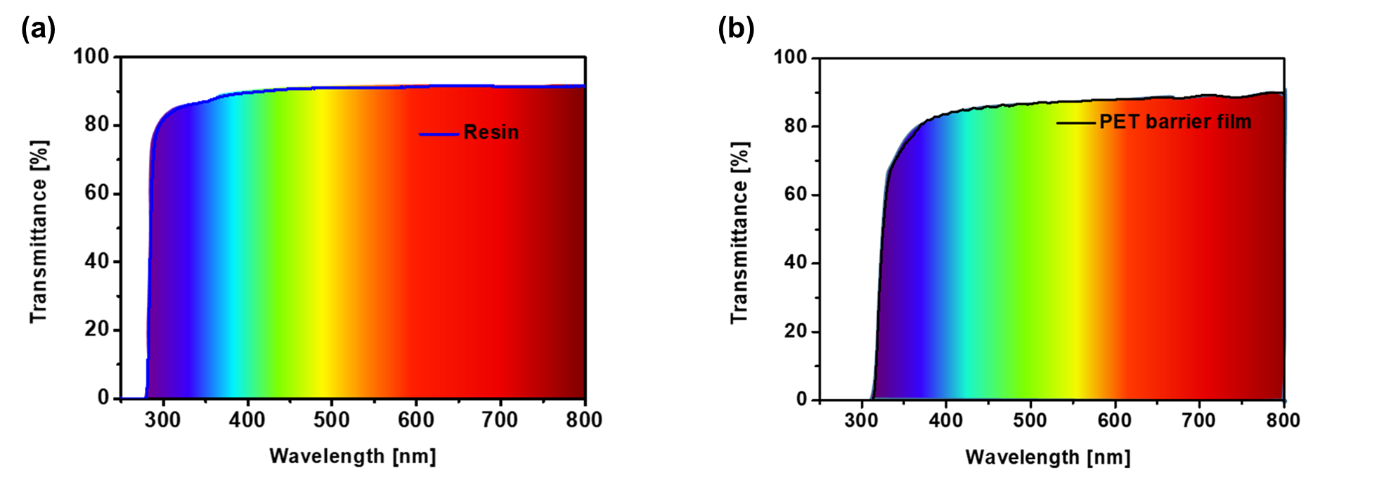


Figure S1. (a) Transmittance spectrum of a resin material coated on a glass substrate. (b) Transmittance spectrum of PET barrier film.

**
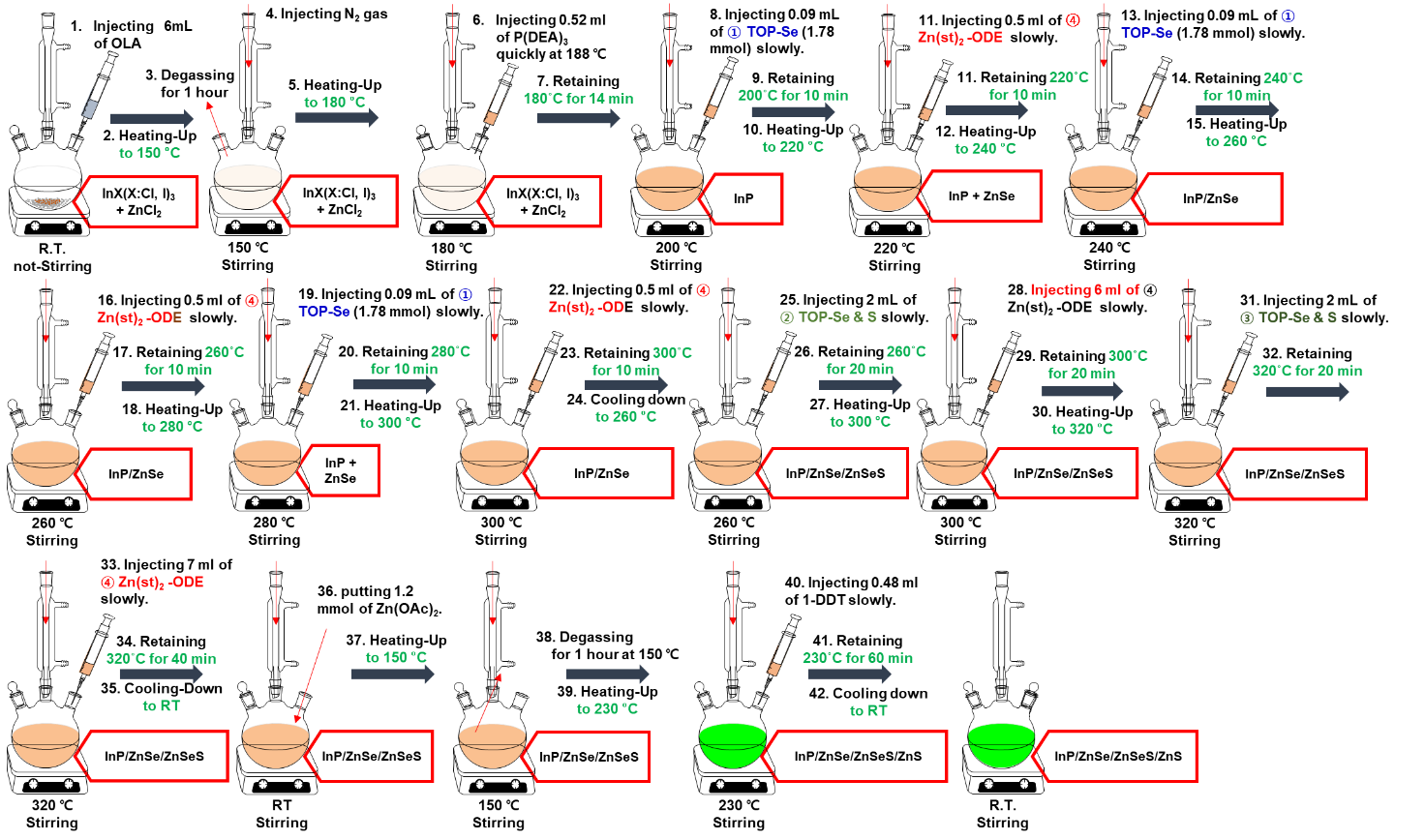
**

Figure S2. Schematic diagram for synthesizing G-InPQD (InP/ZnSe/ZnSeS/ZnS).

**
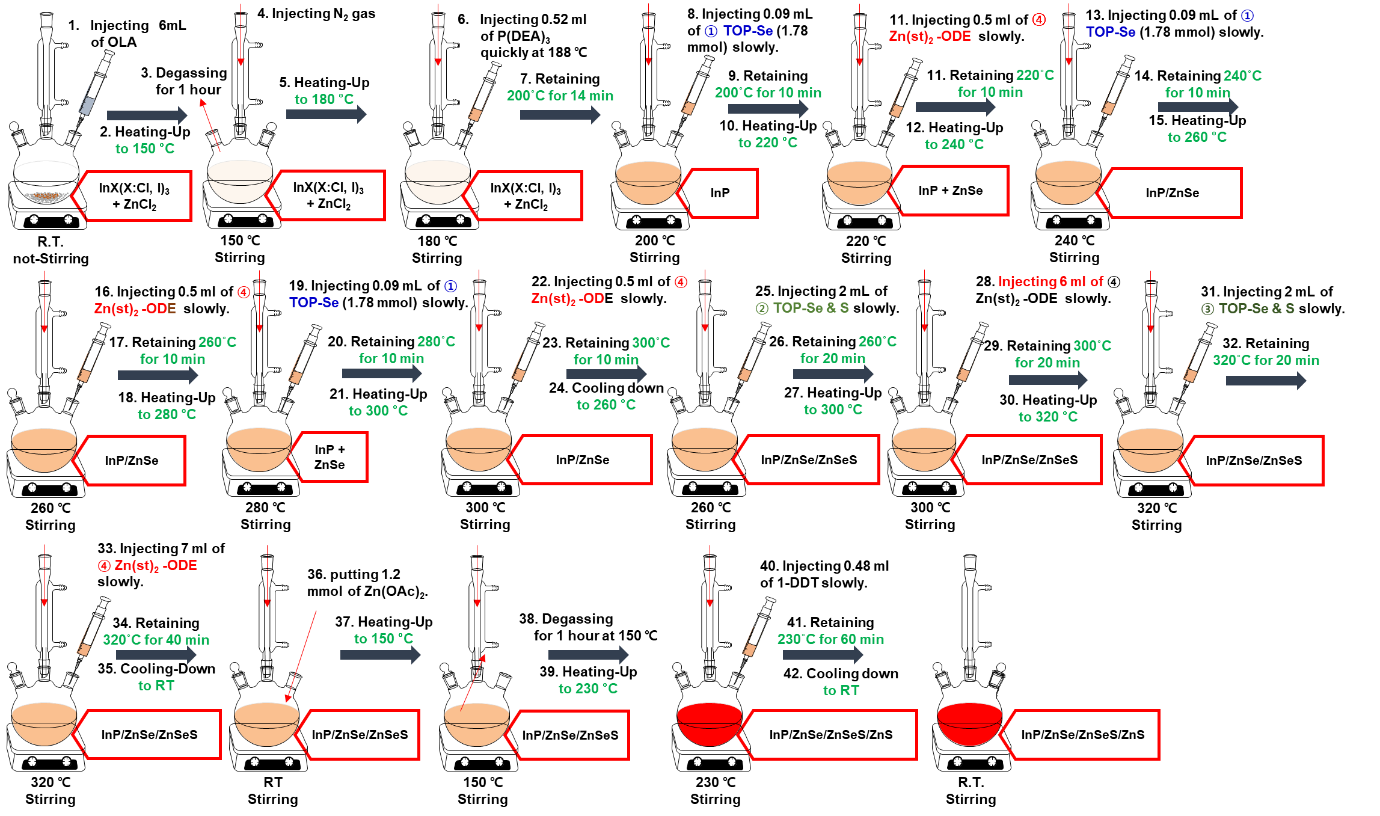
**

Figure S3. Schematic diagram for synthesizing R-InPQD (InP/ZnSe/ZnSeS/ZnS).


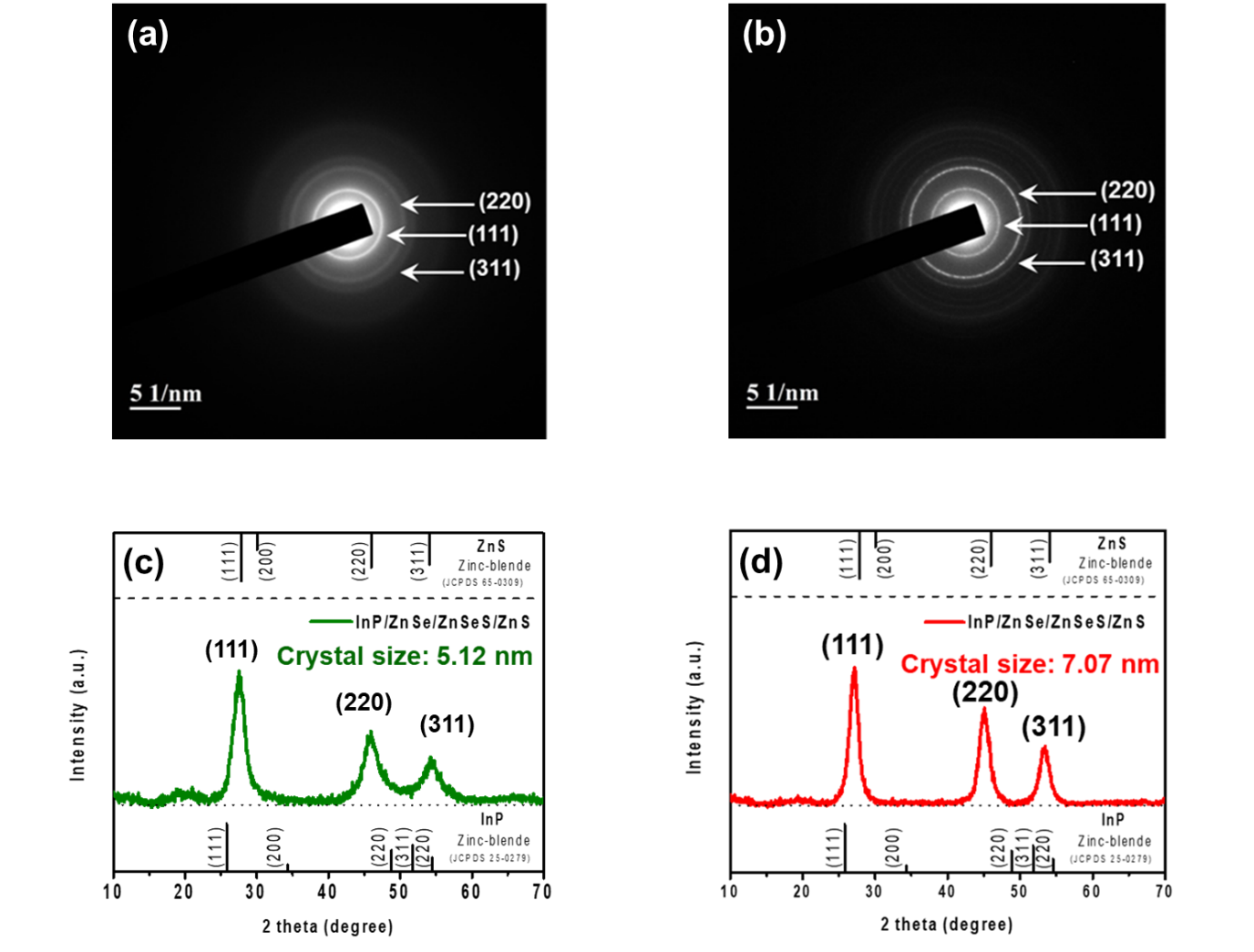
 Figure S4. Crystalline properties of InPZnSe_1-x_S_x_/ZnS QDs. SAED images of (a) G-InPQDs and (b) R-InPQDs; and XRD patterns of (c) G-InPQDs and R-InPQDs, in powder form, showing zincblende crystalline structure.

Figure S5. Time-resolved PL decay curves for the G- and R-InP QDs under 365-nm exitation. The exciton lifetime for G and R-InP QDs were 67.9 and 108.8 ns, respectively, as shown in Table 1. Exciton lifetime (τ) from time-correlated single-photon counting (TCSPC) and Quantum efficiency (QE) are given by the equations:


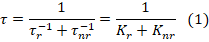

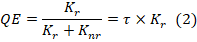


Where *τ_r_*, *τ_nr_*, *K_r_*, and *K_nr_* are radiative recombination time, non-radiative recombination time, radiative rate, and non-radiative rate, respectively. The quantum efficiency of G and R-InP QDs were 85.0 %, and 55.0 %, respectively.

Supplementary Table 1. The exciton lifetime, radiative recombination time, and quantum efficiency for G- and R-InP QDs.

| Samples | G-InP QD | R-InP QD |
| --- | --- | --- |
| τ (ns) | 67.9 | 108.8 |
| τ_r_ (ns) | 1.2 | 0.5 |
| Quantum efficiency (%) | 85 | 55 |


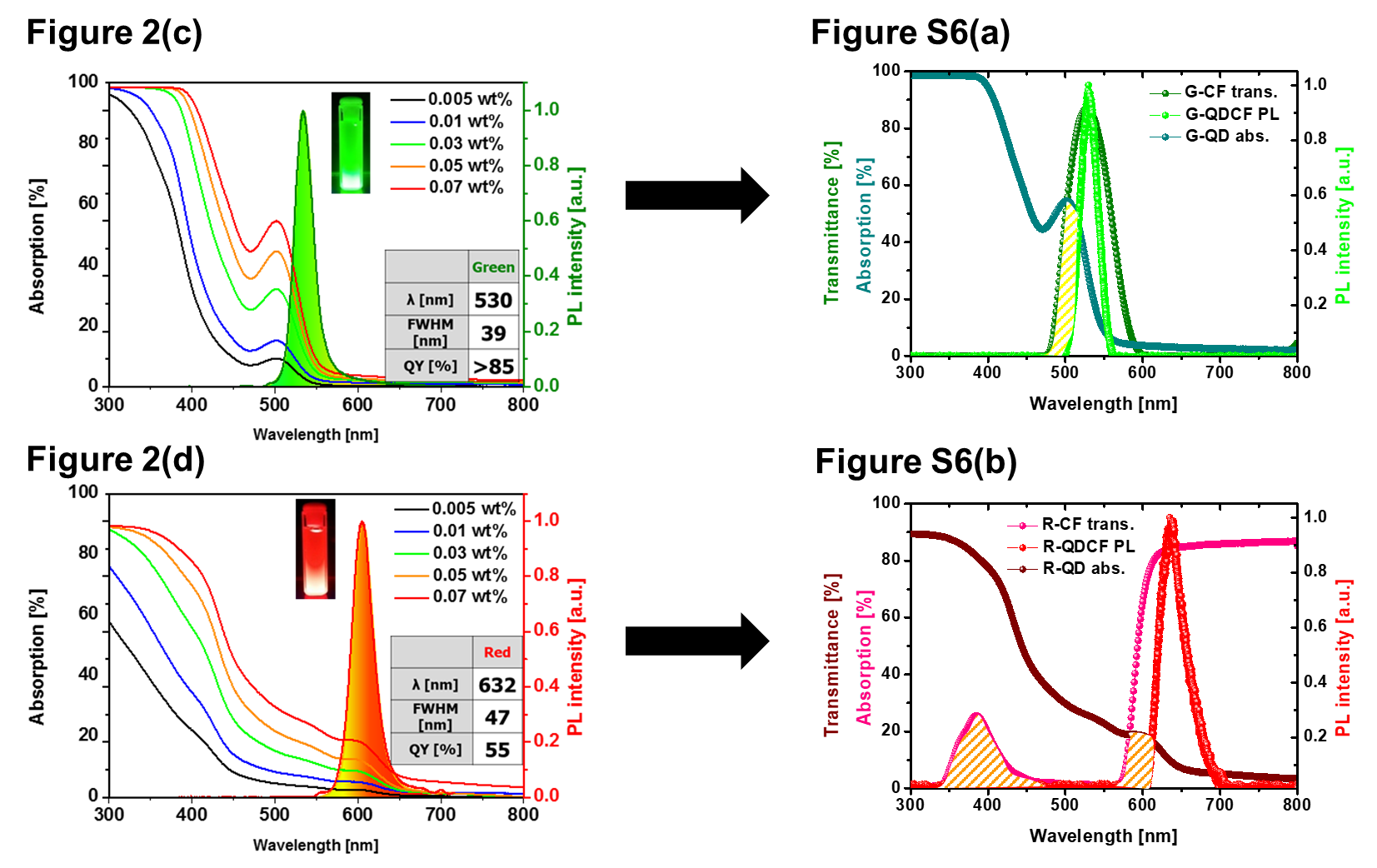


Figure S6. (a) Transmittance spectrum of G-CF(olive color), absorption of G-InP solution(dark cyan color), absorption of G-InP QD functional CF(yellow shadow color), and PL of G-InP QD functional CF(green color). (b) Same as Fig(a) obtained by using R-InP QD.
